# *In Vitro* Differentiation and Maturation of Human Embryonic Stem Cell into Multipotent Cells

**DOI:** 10.4061/2011/735420

**Published:** 2011-08-09

**Authors:** Amer Mahmood, Claudio Napoli, Abdullah Aldahmash

**Affiliations:** ^1^Stem Cell Unit, Department of Anatomy, College of Medicine, King Saud University, Riyadh 11472, Saudi Arabia; ^2^Molecular Endocrinology Laboratory (KMEB), Medical Biotechnology Centre and Department of Endocrinology, University Hospital of Odense, University of South Denmark, 5000 Odense C, Denmark; ^3^College of Medicine, King Saud University, Riyadh 11472, Saudi Arabia; ^4^Department of General Pathology, Division of Clinical Pathology and Excellence Research Center on Cardiovascular Diseases, First School of Medicine, University of Naples Federico II, 80138 Naples, Italy

## Abstract

Human embryonic stem cells (hESCs), which have the potential to generate virtually any differentiated progeny, are an attractive cell source for transplantation therapy, regenerative medicine, and tissue engineering. To realize this potential, it is essential to be able to control ESC differentiation and to direct the development of these cells along specific pathways. Basic science in the field of embryonic development, stem cell differentiation, and tissue engineering has offered important insights into key pathways and scaffolds that regulate hESC differentiation, which have produced advances in modeling gastrulation in culture and in the efficient induction of endoderm, mesoderm, ectoderm, and many of their downstream derivatives. These findings have lead to identification of several pathways controlling the differentiation of hESCs into mesodermal derivatives such as myoblasts, mesenchymal cells, osteoblasts, chondrocytes, adipocytes, as well as hemangioblastic derivatives. The next challenge will be to demonstrate the functional utility of these cells, both *in vitro* and in preclinical models of bone and vascular diseases.

## 1. Introduction

Tissue engineering is an emerging field of research aimed at regenerating functional tissues by combining cells with a supporting substrate. Several different embryonic stem cell lines and adult stem cell sources have been used for this purpose [1–4]; however, some specific cell types may give better results in particular applications. Amongst them, human embryonic stem cells (hESCs) may constitute an important new resource in tissue engineering, mainly due to an extensive differentiation capacity and high proliferative potential. Indeed, many adult organ-specific cells and stem cells show a limited proliferative capacity and lose their differentiated function after long-term *in vitro* culture.

## 2. *In Vitro* Differentiation of ESC

This differentiation only takes place when the correct stimulus is present in the culture media. Although all scientists agree on the potential of hESC, it has also become clear that pluripotency is a double-edged sword; the same plasticity that permits hESC to generate hundreds of different cell types also makes them difficult to control.

## 3. Strategies for Differentiating Human ESC into Three Germ Layers

The basic methods of hESC differentiation are divided into three categories.

1. Direct differentiation as a monolayer on extracellular matrix proteins [5].
2. Differentiation in coculture with stromal cells [6].
3. The formation of 3D spherical structures in suspension culture, termed embryoid bodies (EBs) [7–11].

EB formation is the most common method for initiating differentiation in culture due to its similarity to postimplantation embryonic tissue *in vivo*. It should be noted that EB differentiation does not reconstitute the full array of embryonic development, having no form of polarity or “body plan” All three approaches for hESC differentiation are efficient and have advantages and disadvantages. Each method demonstrates that hESC can differentiate into a broad spectrum of cell types in culture. EB has the advantage of providing a three-dimensional structure, which enhances cell-cell interactions that may be important for some developmental processes. Human ESCs have been successfully differentiated into tissues derived from the three germ layers by the use of all three methods [5, 18, 19].

## 4. *In Vitro* Chondrogenic Differentiation of Human Embryonic Stem Cells

There are three distinct lineages that generate the skeleton: the somites (axial skeleton), lateral plate mesoderm (limb skeleton), and neural crest (skull and face). The skeleton contains three specific cell types: chondrocytes in cartilage, and osteoblasts and osteoclasts in bone. Whereas chondrocytes and osteoblasts are of mesenchymal origin, the osteoclasts are of hematopoietic lineage [20, 21]. 

There are two major modes of bone formation, or osteogenesis, and both involve the transformation of a pre-existing mesenchymal tissue into bone tissue.

1. *Endochondral ossification* is the process by which a cartilage intermediate is formed and replaced by bone.
2. *Intramembranous ossification* is the direct conversion of mesenchymal tissue into bone.

The main difference between these two methods of bone formation is the presence of a cartilaginous phase in endochondral ossification; the mesenchymal cells proliferate and differentiate into prechondrocytes and then into chondrocytes. Chondrocytes are the first skeleton-specific cells to appear during embryonic development. Chondrogenic differentiation of condensed mesenchymal stem cells (MSCs), orchestrated by high-mobility group-box gene Sox9, is the initial event in skeletogenesis, and *Runx2 *controls chondrocyte maturation as well [22, 23].

Chondrogenic differentiation has been stimulated in serum-free media containing exogenous cytokines and growth factors, specifically the TGF-*β* superfamily, under conditions that included 3-dimensional culture. The different studies outlined in Table 1 clearly show that cartilage formation can be achieved by using 3D culture; however, Jukes et al.'s group was the first group to succeed in formation of hyaline chondrocytes from mouse ESCs (mESCs) using a scaffold in conjunction with TGF-*β*3. Furthermore, Jukes' group showed that a complete endochondral ossification can be obtained in combination with matrix mineralization, when hyaline chondrocytes formed *in vitro *are matured *in vivo* [4]. Despite the fact that cartilage has been formed *in vitro*, no study has provided data showing endochondral ossification obtained during *in vitro *culture of either MSCs or ESCs.

Tissue-engineered cartilage can be grown *in vitro* with the use of cell-scaffold constructs and bioreactors [28]. The study by Tigli et al. was designed to investigate the effects of perfusion bioreactors on the chondrogenic potential of engineered constructs prepared from porous silk fibroin scaffolds seeded with hESC-derived MSCs. After four weeks of incubation, constructs cultured in perfusion bioreactors showed significantly higher amounts of glycosaminoglycans (GAGs) (*P* < 0.001), DNA (*P* < 0.001), total collagen (*P* < 0.01), and collagen II (*P* < 0.01) in comparison to static culture. Mechanical stiffness of constructs increased 3.7-fold under dynamic culture conditions, and RT-PCR results concluded that cells cultured in perfusion bioreactors highly expressed (*P* < 0.001) cartilage-related genes when compared with static culture. Distinct differences were noted in tissue morphology, including polygonal extracellular matrix structure of engineered constructs in thin superficial zones and an inner zone under static and dynamic conditions, respectively. The results suggest that perfusion bioreactors can be used to modulate the growth of tissue-engineered cartilage and enhance tissue growth *in vitro.*

## 5. *In Vitro* Differentiation of Human Embryonic Stem Cells intoMesenchymal and Osteogenic Lineages

Osteoblasts are differentiated from multipotent MSCs [29]. This differentiation process is regulated by several cytokines, including BMPs, TGF-*β*, Wnt, and hedgehog [30]. BMP2 is one of the most potent promoters of MSC differentiation into osteoblasts *in vitro *and induces bone formation *in vivo*. Runx2 is the master gene of osteoblast differentiation and directly regulates the expression of osteocalcin and osteopontin, which are two major components of bone matrix [31].

Protocols for directing the differentiation of ESCs to an osteogenic lineage can be divided into at least two approaches (Table 2 and Table 3). The first approach is based on reports from a multistep differentiation of ESCs into an osteoblast lineage. Barberi et al. were the first to differentiate hESCs into a multipotent MSC-like cell population. The hESC-derived MSCs were further differentiated into osteoblasts, adipocytes, chondrocytes, and myoblasts [27]. Other groups achieved an MSC-like differentiation by coculture of ESCs with osteoprogenitor cells (OP9 cells) or the use of growth factors. All protocols sort the MSC population using mesenchymal cell surface markers (Table 2). 

The second approach differentiates ESCs into an osteogenic lineage without any MSC-like step, and all the protocols are more or less the same. The protocols are generally based on two steps; the first step includes EB formation, and the second step consists of adherence culture where the EBs are either disrupted into a single cell population or plated as EBs onto coated tissue culture plates with or without osteogenic differentiation factors. To improve the osteogenic differentiation, different approaches have been used. Kawaguchi et al. added retinoic acid (RA) in the EB step, followed by BMP-2 during the monolayer differentiation [16]; in contrast, Kim et al. cocultured EBs with human primary bone-derived cells to induce osteogenic differentiation [35]. Monolayer differentiation typically requires 21 days, after which the mineralization is observed by either Alizarin red or Von Kossa staining. To demonstrate a successful differentiation of ESCs into active osteoblasts, the expressions of osteoblastic markers are analyzed (Table 3). 

A recent publication describes a new method for producing skeletal muscle progenitor cells that can be further differentiated into mature osteoblasts. This protocol generated myogenic cells by culturing hESCs as EBs in serum-free media containing SB-431542, a small molecule inhibitor of the TGF-*β*/Activin/Nodal signaling pathway [40]. As previously described, EBs lead to the generation of endodermal, mesodermal, and ectodermal lineages. The study showed that SB-431542-mediated inhibition of TGF-*β*/Activin/Nodal signaling led to a decrease in the formation of endodermal cell-types and a dramatic increase in the formation of muscle cell-types, including skeletal muscle, at a fairly high efficiency; 52% of experimental cells expressed MyoD. This study is not only promising in its ability to produce mesodermal skeletal muscle progenitors, but also in the information it provides about the nature of hESC differentiation.

For a protocol to be truly useful for clinical applications it would ideally allow for the directed, homogeneous differentiation of hESCs into the cell type of interest. This means that the best differentiation strategies would avoid random and heterogeneous differentiation steps such as those involved in EB formation. Our recently published studies offer important new directions for the development of future mesodermal differentiation strategies. The first study relies on human EB formation for the initial stages of differentiation [40]. We inhibited TGF-*β*/activin/nodal signaling during EB formation using SB-431542 (SB) in serum-free medium. The inhibition of the TGF-*β* signaling pathway led to selective upregulation of several markers involved in mesoderm induction and myogenic differentiation, as evidenced by enhanced gene expression of TBX6 and Myf5. Explant cultures of EBs in serum-free medium containing SB led to the enrichment of cells with a myogenic phenotype. Further, we demonstrated MSC differentiation of SB-OG cells by the addition of FBS to the culture medium. 

In a second study published by our group, we demonstrate that stromal cells, obtained from hESC cultures by their selective adherence to hyaluronic acid (HA), exhibit characteristics of the hMSC phenotype, including known surface markers, the ability to differentiate into osteoblasts and adipocytes, and formation of ectopic bone when implanted with Hydroxyapatite/Tricalcium phosphate (HA/TCP) subcutaneously in immune-deficient mice [41]. In this study no additional growth factors or cytokines were used for the direct differentiation of hESCs into a homogeneous population of stromal cells, which had the same characteristics as bone-marrow-derived MSCs, including *in vivo* bone formation.

As the potential range of stem cell applications in tissue engineering continues to grow, appropriate scaffolding is necessary to create tightly defined artificial microenvironments for each target organ. These microenvironments determine stem cell fate via control over differentiation. In this study, we examined the effects of scaffold stiffness on embryonic mesenchymal progenitor cell behavior [42]. Mechanically distinct scaffolds with identical microstructure and surface chemistry were produced utilizing core-shell electrospinning. The modulus of core-shell poly(ether sulfone)-poly(*ε*-caprolactone) (PES-PCL) fibers (30.6 MPa) was more than 4 times that of pure PCL (7.1 MPa). The growth of progenitor cells on the two different scaffolds resulted in two distinct populations. The lower modulus PCL fibers provided a more appropriate microenvironment for chondrogenesis, evident by marked upregulation of chondrocytic Sox9, collagen type 2, and aggrecan gene expression, and production of a chondrocyte-specific extracellular matrix glycosaminoglycan. In contrast, the stiffer core-shell PES-PCL fibers supported enhanced osteogenesis by promoting osteogenic Runx2, alkaline phosphatase, and osteocalcin gene expression, as well as alkaline phosphatase activity. These findings demonstrate that the microstructural stiffness of a scaffold and the pliability of individual fibers may play a critical role in controlling stem cell differentiation. This may occur via regulation of distinct cytoskeletal organization and subsequent intracellular signaling events that control differentiation-specific gene expression.

The use of hESCs for tendon tissue engineering has just begun to be explored. Chen et al. placed hESC-derived mesenchymal stem cells (hESC-MSCs) within a knitted silk-collagen sponge scaffold and assessed the efficacy of this construct in promoting tendon regeneration. When subjected to mechanical stimulation *in vitro*, hESC-MSCs exhibited tenocyte-like morphology and expressed the tendon-related gene markers collagen types I and III, Epha4, and scleraxis, as well as other mechanosensory structures and molecules, such as cilia, integrins, and myosin. In ectopic transplantation, the tissue-engineered tendon under* in vivo* mechanical stimulus displayed more regularly aligned cells and larger collagen fibers. This in turn resulted in enhanced tendon regeneration *in situ*, as evidenced by better histological scores and superior mechanical performance characteristics. Furthermore, cell labeling and extracellular matrix expression assays demonstrated that the transplanted hESC-MSCs not only contributed directly to tendon regeneration, but also exerted an environment-modifying effect on the implantation site *in situ*. Hence, tissue-engineered tendon can be successfully fabricated through the seeding of hESC-MSCs within a knitted silk-collagen sponge scaffold followed by mechanical stimulation [43].

## 6. Embryonic-Derived Hemangioblasts

There is a great need for stem cell-derived treatment strategies to stimulate both arteriogenesis and neoangiogenesis during cardiovascular disease [44]. In this regard, hemangioblasts (HSs), which are the precursors of both hematopoietic and endothelial cells, can be generated with well-characterized functional properties by a novel technique from single blastomere-derived stem cells that does not require embryo destruction, thus minimizing ethical and regulatory concerns [45, 46]. This process could have a great therapeutic impact. A recent study reported the effects of intravenous injection of bone marrow cells (BMCs) and HS, supplemented with 1.0% vitamin E, 0.05% vitamin C, and 6% l-arginine, into the ischemic hindlimb of ApoE^−/−^ diabetic and nondiabetic mice [47]. Blood flow was monitored by a laser Doppler blood flow meter, and capillary density was determined in sections of the adductor and semimembranous muscles with an anti-CD31 antibody. BMC or HS alone and BMC plus HS increased blood flow and capillary densities and decreased interstitial fibrosis. These effects were amplified by additional MT, at least in part, through the nitric oxide pathway, reduction of systemic oxidative stress, and macrophage infiltration. Interestingly, telomerase activity was increased by HS treatment. Thus, intravenous HS intervention increased therapeutic angiogenesis in the ApoE^−/−^ diabetic mouse hindlimb. This is also consistent with the evidence that HS from human embryonic stem cells can generate multilayered blood vessels with functional smooth muscle cells (Lu et al. [46]). On the other hand, a very recent study shows that HSs remain epigenetically plastic and require PRC1 to prevent neural gene expression [48], and in another study, when HS derivatives were obtained from induced human pluripotent stem cells, they exhibited limited expansion and early senescence [49]. Further therapeutic strategies may arise from other studies: one that explores the possibility that multipotent progenitor cells are resident in the human fetal aorta wall [49] and another on transplantation of a tissue-engineered, vascularized human cardiac muscle [50].

## 7. Conclusion

Scientists around the world are trying to understand the control of hESC growth and lineage-specific differentiation. These insights enable the reproducible generation of highly enriched cell populations from a number of different lineages. With these tools, we can now begin to test the function of these cell types through the transplantation of highly enriched, well-characterized populations into different preclinical disease models. Access to lineage-specific progenitors for transplantation will allow comparison to more mature populations to determine which stage integrates best into the adult tissue and which ultimately provides the most benefit. The availability of highly enriched cell populations from different lineages also provides an opportunity for cell biologists to interact with tissue engineers to generate culture systems that will more accurately mimic important three-dimensional aspects of organogenesis. Such engineered tissues may be more effective following transplantation and may also support more efficient maturation of the different cell types in culture. With these tools at hand, the therapeutic potential of hESCs is now ready to be tested for example in the field of osteogenic lineages and regeneration of vasculature.

## Figures and Tables

**Table 1:** *In vitro* models for direct differentiation of embryonic stem cells to chondrogenic lineages.

Days in EB formation	Post EB differentiation	Differentiation conditions	Chondrogenic markers studied	Cells line(s)	Reference
5	Encapsulation of EBs in PEG hydrogel for 17 days	The EBs in hydrogel were differentiated in presence of chondrogenic medium (CM) treated with BMP-2 and TGF-*β*1	Type I, II, X collagen, and osteocalcin. Proteoglycans content measurement	Mouse-D3 and human-BG03	[12–15]
N/R	EB cells were seeded on ceramic particles	Cells on the ceramic scaffold was differentiated *in vitro* for 21 days in CM supplemented with TGF-*β*3 and 21 days of *in vivo *maturation	Morphology was studied and proteoglycans content was analyzed	Mouse-IB10, human MSC, and Goat MSCs	[4]
7 RA from day 2	EBs were plated	RA-treated EB outgrowth culture was treated with TGF-*β*3	D15: proteoglycans, Col2a1, Sox9, Col10a1 and MMP13	Mouse-CGR8, E14Tg2a, EFC1	[16]
28	After 4 weeks the EBs were dissociated and self-assembled for additional 4 weeks	EBs were differentiated in chondrogenic medium supplemented with combinations of TGF-*β*1, TGF-*β*3, BMP-2, and IGF-1 (100 ng/mL)	Sox9, Col1a1, Col2a1	Human-BG01V	[17]

Culture medium was high glucose Dulbecco's modified Eagle's medium supplemented with 1–5% foetal calf serum. Factors added for chondrocyte differentiation: ascorbic acid, 50 *μ*g/mL; Dex, 10 or 100 nM; proline, 40 *μ*g/mL; sodium pyruvate, 100 *μ*g/mL; ITS, 50 mg/mL; TGF-*β*, 10 ng/mL; RA, 10^−7^ M; BMP, 10 ng/mL (range, 10–800 ng/mL). *Aggrecan*, *Sox9*, *type II collagen, type X collagen,* and *scleraxis* were analyzed by PCR.

BMP: bone morphogenetic protein; Col: collagen; D: day(s); Dex: dexamethasone; EB: embryoid body; FGF: fibroblast growth factor; IGF: insulin-like growth factor; MMP: Matrix metalloproteases; PEG: poly-ethylene glycerol; RA: retinoic acid; TGF-*β*: transforming growth factor-*β*; OC: osteocalcin.

**Table 2:** Models for differentiation of embryonic stem cells into multipotent mesenchymal stem cells.

Days in EB formation	Differentiation conditions	Selection	Functional assay	Markers studied	Cells line(s)	Reference
None	Coculture of hESC with OP9 cells in serum-containing media	FACS purification with CD73^+^	Differentiation *in vitro* to osteoblasts, chondrocyte, and adipocytes	Cell surface markers: CD29, CD44, CD54, CD73, CD90, CD105 D21: mineralization D17: lipid droplets	Human-H1 and H9	[24]
None	Monolayer direct differentiation in presence of bFGF2 and PDGF AB	FACS purification with CD105^+^ and CD24^−^	Differentiation *in vitro* to osteoblasts, chondrocyte and adipocytes	Cell surface markers: CD29, CD44, CD49a, CD105, CD166 D21: mineralization D14: lipid droplets D21: proteoglycans	Human-H1 and HUES9	[25]
None	Spontaneous monolayer differentiated cells from hESC colonies	Serum (10%)	Differentiation *in vitro* to osteoblasts and adipocytes	Cell surface markers: CD13, CD44, CD71, CD73, CD105, CD166 After 3 weeks: mineralization^a^ D21: lipid droplets	Human-H1	[26]
None	Coculture of hESC with OP9 cells in serum containing media	FACS purification with CD73^+^	Differentiation *in vitro* to osteoblasts, chondrocyte, and myoblasts	Cell surface markers: CD29, CD44, CD54 CD73, CD90, CD105, CD106, CD166, STRO1	Human-H1 and H9	[27]

Culture medium is *α*-minimal essential medium or Dulbecco's modified Eagle's medium. ^a^In this study they did not analyze the main osteoblastic markers by PCR.

bFGF2: basic fibroblast growth factor-2; CD: cluster of differentiation; OP9: mouse stromal cells.

**Table 3:** *In vitro* models for direct differentiation of embryonic stem cells into osteogenic lineages.

Days in EB formation	Differentiation conditions	Selection	Osteoblastic markers studied	Cells line(s)	Reference
1–6	Directed differentiation in presence of osteogenic factors	Serum and osteogenic factors	D21/35: mineralization nodules, ALP, osteocalcin, and type I collagen	Human-H9, mouse ESCs	[32–34]
7 RA from day 2	RA treated EB outgrowth culture was treated with BMP plus osteogenic factors	RA and BMP-4	D12: mineralization	Mouse-CGR8, E14Tg2a, EFC1	[16]
3	Coculture of EBs with primary bone-derived cells (hPBDs) for 14 days, in vivo bone formation with BMP-2	Conditioned media from hPBDs	D7 and 14: mineralization, osteocalcin, collagen I, osteopontin, BSP	Human-CHA3	[35]
5	The differentiation of single cells was initiated with bioactive glass	Serum and osteogenic factors	D21: mineralization, osteocalcin, ALP, and cbfa-1/Runx2	Mouse-E14	[36]
4-5	Single cell suspension of EB was directly differentiated in presence of osteogenic factors	Serum, osteogenic factors and MACS sorting^#^	D21: mineralization, osteocalcin, Cadherin-11, and cbfa-1/Runx2	Human-H1, H9 and Mouse-CEE	[37–39]

Culture medium was *α*-minimal essential medium or Dulbecco's modified Eagle's medium, supplemented with 10–20% foetal calf serum. Factors added for osteogenic differentiation: *β*-gly, 10 mM (range, 2–10 mM); ascorbic acid, 50 *μ*g/mL; Dex, 10 or 100 nM; RA, 10^−7^ M; BMP, 100 ng/mL (range, 10–800 ng/mL). *Cbfa1*, *BSP*, *ALP,* and *OC *were analyzed by PCR.

^#^In the mouse study cells were sorted by Cadherin-11 expression, and cDNA microarray was performed.

ALP: alkaline phosphatase; asc. Acid: ascorbic acid; BMP: bone morphogenetic protein; BSP: bone sialoprotein; *Runx2*: core binding factor *α*1; D: day(s); Dex: dexamethasone; ES: embryonic stem; FGF: fibroblast growth factor; *β*-gly: *β*-glycerophosphate; RA: retinoic acid; OC: osteocalcin.
